# Coverage and beliefs about temephos application for control of dengue vectors and impact of a community-based prevention intervention: secondary analysis from the Camino Verde trial in Mexico

**DOI:** 10.1186/s12889-017-4297-5

**Published:** 2017-05-30

**Authors:** José Legorreta-Soberanis, Sergio Paredes-Solís, Arcadio Morales-Pérez, Elizabeth Nava-Aguilera, Felipé René Serrano de los Santos, Belén Madeline Sánchez-Gervacio, Robert J. Ledogar, Anne Cockcroft, Neil Andersson

**Affiliations:** 1 Centro de Investigación de Enfermedades Tropicales de la Universidad Autónoma de Guerrero, Acapulco, Guerrero Mexico; 2 CIETinternational, New York, NY USA; 3 Department of Family Medicine, McGill University, Montreal, Canada; 4 CIET Trust, Gaborone, Botswana

**Keywords:** Dengue, *Aedes aegypti*, Vector control, Cluster randomised controlled trial, Temephos

## Abstract

**Background:**

Temephos in domestic water containers remains a mainstay of Latin American government programmes for control of *Aedes aegypti* and associated illnesses, including dengue. There is little published evidence about coverage of routine temephos programmes. A cluster randomised controlled trial of community mobilisation in Mexico and Nicaragua reduced vector indices, dengue infection, and clinical dengue cases. Secondary analysis from the Mexican arm of the trial examined temephos coverage and beliefs, and the impact of the trial on these outcomes.

**Methods:**

The trial impact survey in December 2012, in 10,491 households in 45 intervention and 45 control clusters, asked about visits from the temephos programme, retention of applied temephos, and views about temephos and mosquito control. Fieldworkers noted if temephos was present in water containers.

**Results:**

Some 42.4% of rural and 20.7% of urban households reported no temephos programme visits within the last 12 months. Overall, 42.0% reported they had temephos placed in their water containers less than 3 months previously. Fieldworkers observed temephos in at least one container in 21.1% of households. Recent temephos application and observed temephos were both significantly more common in urban households, when other household variables were taken into account; in rural areas, smaller households were more likely to have temephos present.

Most households (74.4%) did not think bathing with water containing temephos carried any health risk. Half (51%) believed drinking or cooking with such water could be harmful and 17.6% were unsure.

Significantly fewer households in intervention sites (16.5%) than in control sites (26.0%) (Risk Difference − 0.095, 95% confidence interval − 0.182 to −0.009) had temephos observed in their water; more households in intervention clusters (41.8%) than in control clusters (31.6%) removed the applied temephos quickly. Although fewer households in intervention sites (82.7%) compared with control sites (86.7%) (RD -0.04, 95% CI -0.067 to −0.013) agreed temephos and fumigation was the best way to avoid mosquitoes, the proportion believing this remained very high.

**Conclusion:**

Coverage with the government temephos programme was low, especially in rural areas. Despite an intervention encouraging non-chemical mosquito control, most households continued to believe that chemicals are the best control method.

**Trial registration:**

ISRCTN:27581154.

**Electronic supplementary material:**

The online version of this article (doi:10.1186/s12889-017-4297-5) contains supplementary material, which is available to authorized users.

## Background

The recent epidemic of Zika has highlighted the failure of chemical control of the *Aedes aegypti* mosquito, the vector for zika, as well as for dengue and chikungunya. A report of a World Health Organisation (WHO) meeting of experts noted the lack of “evidence that any recent vector-control interventions, including massive spraying of insecticides, have had any significant effect on dengue transmission” [1]. The number of dengue cases in Brazil increased between 1990 and 2015, despite a strategy to control *Aedes aegypti* based on use of insecticides and larvicides, and authors have called for prevention to focus instead on improvement of water supply to avoid the need to store water [2]. WHO recommends Integrated Vector Management for control of the dengue vector, which combines both chemical and non-chemical control methods, prioritising environmental control over use of chemicals [3].

Nevertheless, dengue control in most Latin American countries continues to depend on the use of the organophosphate pesticide temephos in domestic water stores. In Mexico, the dengue action plan 2007–2012 aimed to achieve universal and quality coverage with temephos in order to reduce the burden of dengue illness [4]. The dengue action plan 2013–2018, while continuing to be based on chemical control of the dengue vector, restricted the prevention programme, including application of temephos, to the 100 municipalities with the highest rates of transmission and dengue illness [5].

With the extensive use of temephos globally, there is evidence of vector resistance. There are continuing reports of *Aedes aegypti* temephos resistance in many Latin and South American countries, including Brazil [6], Cuba [7], El Salvador [8], Argentina [9], Bolivia [10], Venezuela [11], Peru [12], Dominican Republic [13], and Colombia [14]. Developing resistance leads to higher dose applications of temephos for vector control. Even in the absence of resistance, water use practices limit the effectiveness of temephos, with frequent re-filling of water containers reducing residual larvicidal effects [15].

There remains uncertainty about potential human health effects of exposure to temephos applied into household water supplies. Studies have demonstrated that temephos has cytostatic and genotoxic effects on human cells in vitro [16, 17]. A small study in India of 70 workers occupationally exposed to a mixture of pesticides, including temephos, reported evidence of DNA damage, decreased anti-cholinesterase activity and abnormal liver enzymes [18]. One tiny study in 19 male prisoners in 1967 reported no inhibition of cholinesterase activity in the plasma or in erythrocytes with doses up to 4.27 mg/kg body weight for 5 days [19]. A 1968 report described adding temephos to community drinking water storage containers for a community of about 2000 people; the authors reported no clinical manifestations related to the exposure and no detectable reductions blood levels of cholinesterase among 38 community residents followed during the 19-month experiment [20].

A WHO meeting to consider toxicity of temephos added to drinking water concluded that at the recommended maximum of 1 mg/l, temephos in drinking water would not be harmful to an adult drinking two litres per day, but recommended to consider using alternative sources of drinking water for small children and bottle-fed infants for a period after an application of temephos [21]. WHO classifies temephos as unlikely to cause acute hazard under conditions of normal usage [22].

A recent systematic review of community-effectiveness of temephos intervention studies (alone or in combination with other interventions) concluded that temephos application, as a single intervention, reduced entomological indices but there was no evidence that it reduced dengue transmission [23]. Outside of the research context, temephos might be less effective when applied as part of a routine control programme. A recent cluster randomised controlled trial of community participation for dengue prevention in Mexico and Nicaragua reported, as a secondary finding, an association between the presence of temephos in household water containers and *higher* levels of serological evidence of dengue infection [24]. The association was not explained by authorities applying temephos after a case of dengue was reported; it persisted when households reporting a clinical case of dengue were excluded from the analysis. The authors speculated that the increased risk of dengue infection with temephos presence might be because households knowing they had temephos in their water containers could be demotivated from taking physical measures to deal with mosquitoes and potential mosquito breeding sites.

The effectiveness of vector control using temephos will be reduced further if actual coverage achieved is less than intended, especially if the programme fails to reach households with a higher likelihood of having dengue cases. We have found only two articles, from Thailand and Malaysia, reporting on coverage with a routine temephos application programme [25, 26]. The present article uses data from the impact survey of the Mexican arm of the Camino Verde dengue prevention trial [24] to estimate coverage with the routine government temephos application programme, to examine the factors associated with this coverage, and to examine household beliefs about temephos and how to control mosquitoes. We were also able to examine the impact, if any, of the community-mobilisation intervention on temephos coverage and household beliefs.

## Methods

This article is based on a secondary analysis of data collected between December 2012 and January 2013 during the impact survey of the Mexican arm of a cluster randomised controlled trial of community mobilisation for dengue prevention. The trial methods and findings are described in detail elsewhere [24]. In brief, after stratification by vector levels in the baseline survey, the trial randomly allocated 90 clusters (census enumeration areas, each of about 140 households) in the three coastal regions of Mexico’s Guerrero State to either the intervention (45) or control (45) group, and implemented a community-based intervention for chemical-free control of the dengue vector *Aedes aegypti* in the intervention clusters. Normal government dengue prevention efforts continued in all communities, including the programme of application of the insecticide temephos in household water containers [4]. The intervention included household visits from neighbourhood teams (*brigadistas*) and community activities to educate people about the life cycle of the mosquito and support them in efforts to reduce breeding sites in households and elsewhere on the community [24, 27, 28]. The trial achieved significant reductions in all vector indices, in dengue infection measured by saliva dengue antibody serology, and in self-reported dengue cases [24].

In the 2012/2013 impact survey, trained field teams conducted a household survey in the 90 clusters, administering a questionnaire and conducting an entomological survey of water containers in the households. While inspecting the household water containers, they noted whether plastic sachets of temephos were present, as well as collecting any larvae or pupae for later entomological identification. Questions about temephos coverage in the household questionnaire included: how many temephos-application visits the household had received in the last 12 months; how long ago temephos was most recently applied; and how long the household left this temephos in their water storage containers. The questionnaire asked if the respondent thought bathing in water containing temephos was harmful to health and in what way; and if they thought drinking or cooking with water containing temephos was harmful and in what way. Interviewers asked respondents if they agreed with the statement “Application of temephos and/or space fumigation are the best way to avoid mosquitos”.

In the household survey, we also collected and categorised information about socio-economic variables including: household structure (permanent v semi-permanent or temporary); access to tap water (daily v less frequent or no access); language spoke at home (Spanish only, or an indigenous language); registration in the government *Oportunidades* programme (which supports poor households, for example to send children to school); sex of the respondent; household size (less than five members v five or more); employment of the household head (employed or not); and education of the household head (4 years of education or more v less than 4 years).

### Analysis

Trained operators used the Epidata programme to enter data twice, with validation to minimise keystroke errors. Analysis relied on CIETmap [29], which provides a user-friendly interface with the R statistical programming language.

We established three operational definitions of household temephos coverage, bearing in mind the government policy that temephos should be applied every 2 months. The three definitions of coverage were: reported five or more visits for temephos application by a team from the government temephos programme in the last 12 months; last reported temephos application within the last 3 months; and the presence of temephos observed in at least one water container. Among all households, we examined factors potentially associated with temephos coverage in bivariate and then multivariate analysis, using the Mantel-Haenszel procedure [30] with a cluster-adjustment [31]. We included in the initial multivariate model those factors significantly associated with the coverage outcome in bivariate analysis. We report significance of associations using the Odds Ratio (OR) and cluster-adjusted 95% confidence interval (95% CIca) of the OR.

We examined the impact of the trial intervention on temephos coverage, beliefs about temephos health effects, and agreement that temephos and/or fumigation was the best method to avoid mosquitos. In this analysis we calculated the Risk Difference (RD) of proportions in intervention and control clusters, and the cluster-adjusted 95% confidence interval of the RD.

## Results

The survey teams interviewed 10,491 households: 3426 in Acapulco, 3425 in Costa Grande, and 3640 in Costa Chica. Most of the household respondents were women (80.4%; 2050/10,453) and over half the households were in rural communities (56.1%; 5886/10,491). Most households reported speaking Spanish only (89.8%; 9352/10,416); a few reported speaking indigenous languages. Most of the households said they had access to tap water (85.4%; 8947/10,478) but only a minority of these had a daily supply of tap water (39.1%; 3489/8913); overall, 33.4% (3488/10434) of households had daily access to tap water. Almost half (48.6%; 5082/10,456) of the households were beneficiaries of the *Oportunidades* programme. Now called *Prospera* **,** this is a Mexican government programme of cash transfers to mothers to encourage them to send their children to school and to health centres. Less than half the households (39.2%; 4019/10,256) reported participating in community activities to control mosquito breeding sites.

### Coverage with the temephos programme

**Fig. 1:**
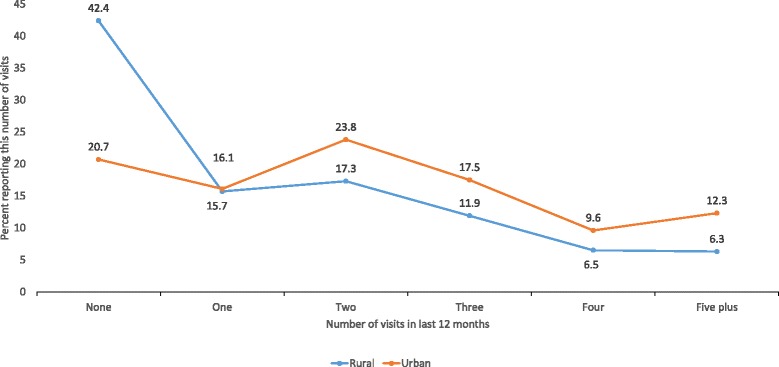
Number of visits from temephos programme officers within the last 12 months, as recalled by households

Figure 1 shows the coverage of the temephos programme in urban and rural communities, according to the recall of the household respondents of the number of visits from programme officers within the last 12 months. The programme is supposed to visit every 2 months to apply temephos, in which case households should report at least five visits in the last 12 months. Few households reported this number of visits: 12.3% (543/4398) in urban areas and 6.3% (354/5679) in rural areas. The proportion of households reporting at least five visits was significantly higher in urban areas (OR 2.11; 95% CIca 1.36 – 3.29). Some four out of ten households in rural areas (42.4%, 2406/5679) and two out of then in urban areas (20.7%, 909/4398) reported no visits at all within the last 12 months.

The survey took place at the beginning of the dry season, in November and December 2012, and less than half the households (42.0%, 3773/8978) said that they had temephos placed in their water containers less than 3 months previously. The proportion reporting recent temephos application was higher in urban sites (49.1%, 1994/4060) than in rural sites (36.2%, 1779/4918).

In answer to a separate question about retention of the temephos most recently placed in water containers, some 36.8% (3167/8604) of the households said they had never had temephos in their water or they had removed the most recently inserted temephos after less than 1 month. This proportion was higher in rural areas (42.2%, 1963/4653) than in urban areas (30.5%, 1204/3951). In some cases, households reported removing the temephos after only a matter of hours or days.

Overall, the field teams observed the presence of temephos in 20.6% (10,667/51825) of containers in which it could be inserted: 30.5% (6643/21815) of containers in urban areas and 13.4% (4034/30010) of containers in rural areas. They observed temephos in at least one container in 21.1% (2101/9937) of households: 30.4% (1292/4251) in urban households and 14.2% (809/5686) in rural households.

### Factors related to temephos coverage

In bivariate analysis of factors potentially related to reported temephos application within the last 2 months, urban residence and male sex of the household respondent were significantly associated with reported recent application of the pesticide (Table 1). Sex of respondent was no longer significantly related to the outcome, when area of residence was taken into account.

**Table 1:** Bivariate analysis of factors associated with reported placing of temephos in household water containers within the last 3 months, among 8978^a^ households in 2012

Potential associated factor	Levels	With temephos *n* (%)	Without temephos *n* (%)	OR	95% CIca
All households		21.1% (2101/9937)	78.9% (7836/9937)		
House structure	Permanent	2385	3074	1.19	0.98–1.45
	Semi-permanent/temporary	1373	2108		
Language spoken at home	Indigenous language	349	597	0.79	0.50–1.26
	Spanish only	3384	4583		
Area of residence	Urban	1994	2066	**1.70**	**1.07**–**2.70**
	Rural	1779	3139		
Region	Costa Grande & Costa Chica	2609	3492	1.10	0.66–1.82
	Acapulco	1164	1713		
Oportunidades programme	Participating	1807	2581	0.93	0.72–1.21
	Not participating	1954	2608		
Household size	Less than five members	2234	3089	0.99	0.89–1.11
	Five or more members	1539	2115		
Sex of household respondent	Female	2997	4254	**0.85**	**0.74**–**0.97**
	Male	769	927		
Education of household head	4 years or more	2248	3327	1.06	0.91–1.22
	Less than 4 years	1279	1838		
Employment of household head	Working	3340	4792	0.92	0.74–1.13
	Not working	296	378		
Tap water supply	Daily	1273	1744	1.01	0.71–1.44
	Less frequent or no supply	2481	3437		

*OR* Odds Ratio

*95% CIca* cluster adjusted 95% confidence interval

Bold font indicates associations significant at the 5% level

^a^960 household respondents did not know if temephos had been placed in their water containers, and the data about temephos application were missing in 553 records

Table 2 shows the bivariate analysis of potential associations with the observed presence of temephos in at least one water container in the household. Urban households, with a permanent structure, and with less than five members, were more likely to have temephos present. Household where the respondent was female and participating in the Oportunidades programme were less likely to have temephos present. In multivariate analysis there was interaction and we therefore constructed separate models for urban and rural households. In rural areas, the only factor remaining significantly associated with temephos presence was household size, with smaller households more likely to have temephos present (OR 1.31, 95% CIca 1.10–1.56), while in urban areas no other factors remained to the present of temephos.

**Table 2:** Bivariate analysis of factors associated with presence of temephos in at least one household water container, as observed by the field workers among 9937 households in 2012

Potential associated factor	Levels	With temephos *n* (%)	Without temephos *n* (%)	OR	95% CIca
All households		21.1% (2101/9937)	78.9% (7836/9937)		
House structure	Permanent	1356	4591	**1.29**	**1.03–1.62**
	Semi-permanent/temporary	735	3212		
Language spoken at home	Indigenous language	180	825	0.80	0.53–1.21
	Spanish only	1895	6966		
Type of community	Urban	1292	2959	**2.63**	**1.63–4.25**
	Rural	809	4877		
Region	Costa Grande & Costa Chica	1423	5387	0.95	0.53–1.73
	Acapulco	678	2449		
Oportunidades programme	Participating	863	4052	**0.65**	**0.49–0.86**
	Not participating	1227	3760		
Household size	Less than five members	1315	4596	**1.18**	**1.05–1.33**
	Five or more members	786	3239		
Sex of household respondent	Female	1619	6342	**0.79**	**0.69–0.90**
	Male	476	1466		
Education of household head	4 years or more	1353	4959	1.07	0.88–1.29
	Less than 4 years	721	2817		
Employment of household head	Working	1898	7220	0.84	0.69–1.03
	Not working	178	572		
Tap water supply	Daily	733	2609	1.07	0.72–1.60
	Less frequent or no supply	1355	5184		

*OR* odds ratio

*95% CIca* cluster adjusted 95% confidence interval

Bold font indicates associations significant at the 5% level

### Opinions and beliefs about temephos and vector control approaches

Most households (74.4%; 7793/10474) did not think that bathing with water containing temephos carried any health risk. A few (16.9%, 1774/10474) thought it could damage health or were unsure about it (8.7%, 907/10474). The most common health concern cited was skin problems (83.9%; 1237/1475). Respondents expressed more concerns about drinking or cooking with water containing temephos. About a third (31.4%; 3272/10416) considered this was not harmful to health, while half (51%; 5314/10416) believed it could be harmful and 17.6% (1830/10416) were unsure. Health concerns included: gastro-intestinal problems (35.4%; 2556/7219); infection, 6.7% (486/7219); poisoning, 4.6% (334/7219); and allergies, 3.7% (268/7279).

The great majority of household respondents (84.7%; 8782/10370) agreed that the temephos programme (*abatizando*) and area fumigation were the best way to avoid mosquitoes. None of the socio-economic variables we examined was associated with this prevalent belief.

### Impact of the Camino Verde intervention

The proportion of households reporting coverage with the temephos programme (either at least five visits within the last 12 months or temephos placed in their water within the last 2 months) was not significantly different between intervention and control sites (Table 3). However, the proportion of households where the field team observed temephos in at least one water container was significantly lower in intervention sites (16.5%) than in control sites (26.0%) (Table 3). This may be related to less retention of temephos in water among households in intervention sites (41.8% removed the temephos after less than 1 month in intervention sites compared with 31.6% in control sites), although this difference was not significant at the 5% level.

**Table 3:** Temephos coverage, and beliefs of household respondents in 45 intervention and 45 control sites

Variable	Intervention sites Percent (fraction)	Control sites Percent (fraction)	RD and 95% CIca
*Temephos coverage*			
Temephos placed in water within last 2 months	39.4 (1804/4581)	44.8 (1969/4397)	−0.054 (−0.167 to 0.059) *P* = 0.49
5+ visits by temephos team within last 12 months	10.6 (543/5107)	7.2 (358/4970)	−0.034 (−0.004 to 0.072) *p* = 0.06
Temephos present in at least one water container	16.5 (839/5088)	26.0 (1262/4849)	**−0.095 (−0.182 to − 0.009)** ***P*** **= 0.02**
Temephos retained <1 month or no temephos	41.8 (1831/4382)	31.6 (1336/4222)	0.102 (0.019 to 0.18) *p* = 0.095
*Beliefs and opinions*			
Bathing in water with temephos is harmful	22.2% (1086/4884)	14.7% (688/4683)	**0.075 (0.049 to 0.10)** ***p*** **= <0.0001**
Drinking or cooking with water with temephos is harmful	63.4% (2832/4469)	60.3% (2482/4117)	0.031 (−0.020 to 0.051) *p* = 0.197
Temephos and fumigation is the best way to control mosquitos	82.7% (4377/5291)	86.7% (4405/5079)	**−0.04 (−0.067 to − 0.013)** ***p*** **= 0.004**

*RD* risk difference

*95% CIca* cluster adjusted 95% confidence interval

Bold font indicates associations significant at the 5% level

Table 3 also shows the views of households about health effects of temephos. A significantly higher proportion of households in intervention sites thought that bathing in water with temephos could be harmful. However, there was no difference in the proportion of households thinking that drinking or cooking with temephos-containing water could be harmful between intervention and control sites.

The proportion of households believing that temephos application and fumigation was the best method to control mosquitos was significantly lower in intervention sites than in control sites, but still remained very high at 82.7%.

## Discussion

### Coverage with temephos programme

Our three measures of household coverage (five or more visits from the temephos team in the last 12 months, temephos last placed in household water less than 3 months ago, and temephos observed in at least one water container during the survey) all indicate low coverage of the government temephos application programme in 2012, especially in rural areas. Only 6% of rural households said they had been visited five times or more and four out of ten said they had not been visited at all in the last year. The situation was slightly better in urban areas, where only two out of ten households had been missed altogether and 12% reported five visits or more. Whatever the number of programme visits across the year, less than half the households, at the beginning of the dry season, reported having temephos placed in their water containers within the last 3 months (that is, during the peak dengue season). And the field teams observed temephos (in *any* water container) in even fewer households: 30% in urban sites and 14% in rural sites. This is far from universal coverage, which was the aim of the programme in 2012.

We can compare our estimates of coverage with the government dengue control programme figures for January to December 2012 reporting the number of household visits made by the vector control teams in the three regions. Based on these figures, the census populations of the regions, and estimated household size, some 18% of households had three visits during the year in Costa Grande, 6% in Costa Chica, and 37% in Acapulco (Dr Rufino Silva Dominguez, Personal Communication; Additional file 1). From the government programme figures, across the three regions, about 26% of households had three visits during the year. This figure is quite close to the overall figure from the survey of temephos observed in at least one container in 20.6% of households. There are some differences between regions, with a relatively high proportion of households in Costa Chica having temephos observed in the survey, despite a lower number of visits reported by the government programme in this region. This might reflect that households in this region retain the applied temephos in their water containers for longer than in the other regions.

We found few associations between temephos coverage and socio-economic variables. The main factor was area of residence, with much higher coverage in urban areas. Within rural areas, smaller households were more likely to have temephos present, although such households have been found to have a higher risk of self-reported dengue cases, perhaps because of better recognition of the condition [32]. Temephos application in response to recognition of a clinical case of dengue might explain the association with household size. There is evidence that households without a regular water supply have higher entomological indices [33] and higher rates of dengue cases [34], and perhaps they should be especially targeted by the temephos programme, but we found no association between water supply and temephos coverage.

There is surprisingly little published evidence about coverage with routine government temephos application programmes. A cross-sectional survey of 966 households in Thailand reported higher use of temephos in rural (60%) than in urban households (25%) in the last 12 months; with 16% of rural households and 7% of urban households treating with temephos more often than quarterly [25]. This contrasts with our much higher coverage in urban areas. Unlike in Mexico, where government officers place temephos in household water containers, in Thailand, temephos is delivered to households and household members are responsible for placing the chemical in their water containers; the system of distributing temephos is different between urban and rural sites. Less than half (47%) of 2512 respondents to a telephone survey in Malaysia reported putting temephos in their water containers to prevent mosquitoes breeding there, while most (75%) reported covering their water containers [26].

The coverage of temephos application achieved can make a difference. A study in Clorinda, Argentina, of the impact of a programme of city-wide household inspections and temephos application found that coverage with temephos varied between areas and that reduction in larval indices was related to the proportion of households visited and treated [35].

### Perceptions about temephos and mosquito control

About a quarter of household respondents in our study considered bathing in water containing temephos was, or might be, damaging to health, but the majority (69%) believed that drinking or cooking with water containing temephos posed, or might pose, a health risk. Other authors have reported public concerns about adverse health effects of temephos. A qualitative study in Thailand reported a common perception that temephos is a harmful chemical that should not be placed in drinking water [36]. Interviews with 54 people in Cuba living in houses where *Aedes aegypti* were detected suggested that some of them were convinced that water containing temephos was not safe to drink [37]. Focus groups and in-depth interviews in Peru suggested that discolouration and bad taste, rather than health concerns, were the main reason for refusing temephos addition to drinking water [38].

The great majority of household respondents in our study agreed that fumigation and temephos application was the best way to avoid mosquitoes. This belief may reflect the strong reliance on these methods by the government vertical programme for dengue control over many years. The government programme typically intensifies activities such as temephos application and fumigation at the height of the annual dengue season, under pressure from the media and communities themselves. From this point, the number of dengue cases and mosquitoes will reduce in any case, from reduction of susceptibles and with the advent of the dry season with less vector breeding, but the reduction is likely to be associated in people’s minds with the visible intensification of chemical-based actions. Other authors have reported strong public belief in the efficacy of temephos and, in particular fumigation, for mosquito control. A study in three communities in Mexico, using in-depth interviews, reported a prevailing belief in the government programme of using temephos and fumigation to control dengue, despite some concerns about frequency, safety, and efficacy of temephos application [39]. The authors considered that this could be related to the long-standing use of the same vertical programme for dengue prevention, or because knowledge and beliefs did not favour a change towards more individual responsibility for prevention. Focus groups in Thailand considered that insecticide spraying was the best way to control mosquitoes [36]. However, in Peru, some groups were reluctant to agree to fumigation because they thought it was ineffective [38].

### Impact of Camino Verde intervention

In the Camino Verde trial, the government dengue control programme, including temephos application, continued in both intervention and control sites, and the intervention was not designed to detract from this programme [24]. However, we did detect a reduction in presence of temephos in intervention clusters compared with control clusters in the impact survey, perhaps related to the larger proportion of households removing applied temephos after only a short time. A greater proportion of households in intervention sites believed bathing with water containing temephos could be harmful. And there was a significant, small, reduction in the proportion of respondents who agreed temephos and fumigation was the best method to avoid mosquitoes, although this figure remained very high. Perhaps the intervention focus on non-chemical means of vector control meant that some people began to question the reliance on temephos and other chemicals. It is possible that in some clusters, members of the local mobilisation teams (*brigadistas*) may have specifically discouraged the use of temephos, while encouraging households to use alternative methods for preventing mosquito breeding. The materials used by the brigadistas concerned the mosquito life-cycle and how this could be interrupted by non-chemical means, but they did not specifically discourage the use of temephos. In some intervention communities it became popular to use larvivorous fish to reduce mosquito breeding [40]. These fish cannot survive in water containing temephos, and households with fish present in any water container were indeed much less likely to have temephos found in any water container (OR 0.26, 95% CIca 0.18–0.36).

But it is clear that, despite the intervention, most households continued to believe that a programme outside their own control is what is needed to control the dengue vector. The report of the Camino Verde trial noted that significantly more households in intervention clusters than control clusters believed that communities themselves could control dengue, but nevertheless this proportion was less than 50% even in intervention clusters [24].

### Limitations

The estimate of temephos coverage relying on recall of visits over the last 12 months may not be entirely reliable, perhaps tending to under-estimate visits that happened longer ago. However, the low coverage reported by households was also reflected in figures from the government vector control programme. Recall of the timing of the last application of temephos is likely to be more reliable, and the direct observation of temephos in the water containers does not rely on recall. The question about the best method for mosquito control was not an open question and only one method was mentioned. The question was worded “Many people believe that the best method to avoid mosquitoes is to use temephos and fumigation. Do you agree this is the best method?” This approach might have inflated the apparent support for temephos and fumigation as the best method for mosquito control.

## Conclusion

Coverage with the routine government temephos programme was low, especially in rural areas, and this is likely to reduce its impact as the mainstay of government dengue prevention efforts. There was some evidence that the trial intervention of community mobilisation for vector control led to reduced reliance on temephos, but nevertheless nearly all households in intervention sites continued to believe that temephos and fumigation is the best way to control mosquitoes, and many were not confident that they could achieve mosquito control through their own actions. This has implications for the sustainability of activities initiated during the trial period.

## Additional file

Additional file 1: Table of coverage estimated from government figures for visits by temephos programme, and survey estimates of coverage, 2012. (PDF 43 kb)
