# Supplementary material for: Coverage and beliefs about temephos application for control of dengue vectors and impact of a community-based prevention intervention: secondary analysis from the Camino Verde trial in Mexico
Source: BMC Public Health. 2017 May 30;17(Suppl 1):426. doi: 10.1186/s12889-017-4297-5 (PMC5506576; doi:10.1186/s12889-017-4297-5)
Supplement: Additional file 1: — Table of coverage estimated from government figures for visits by temephos programme, and survey estimates of coverage, 2012. (PDF 43 kb) [file 12889_2017_4297_MOESM1_ESM.pdf]

Additional file 1. Coverage estimated from government figures for visits by temephos programme, and survey estimates of coverage, 2012

	2010 census figures		Government programme figures (Jan-Dec)			Survey figures (% of hh)	
				Proportion of hh visited (%)		Visited within last 12m	Temephos observed
Region	Population	No. hh*	No. visits	If 1 visit/hh	If 3 visits/hh		
Costa Grande	413,793.0	82,758.6	**45,655**	55.2	18.4	68.1	18.7
Costa Chica	428,501.0	85,700.2	**14,012**	16.4	5.5	64.0	23
Acapulco	789,971.0	197,492.8	**221,561**	112.2	37.4	69.5	21.7
Total		365,951.6	**281,228**	76.8	25.6	67.1	20.6

* Based on estimated household size =5 in Costa Grande and Costa Chica and =4 in Acapulco
The figures in **bold** are from the government programme database, from 1 January to 31 December 2012

hh= household
